# Unraveling the Dichotomy of Enigmatic Serine Protease HtrA2

**DOI:** 10.3389/fmolb.2022.824846

**Published:** 2022-02-03

**Authors:** Ayon Chakraborty, Roshnee Bose, Kakoli Bose

**Affiliations:** ^1^ Integrated Biophysics and Structural Biology Lab, ACTREC, Tata Memorial Centre, Navi Mumbai, India; ^2^ Homi Bhabha National Institute, BARC Training School Complex, Mumbai, India

**Keywords:** HtrA2, allostery, PDZ domain, enzyme, apoptosis, cancer, neurodegradation

## Abstract

Mitochondrial high-temperature requirement protease A2 (HtrA2) is an integral member of the HtrA family of serine proteases that are evolutionarily conserved from prokaryotes to humans. Involvement in manifold intricate cellular networks and diverse pathophysiological functions make HtrA2 the most enigmatic moonlighting protease amongst the human HtrAs. Despite perpetuating the oligomeric architecture and overall structural fold of its homologs that comprises serine protease and regulatory PDZ domains, subtle conformational alterations and dynamic enzymatic regulation through the distinct allosteric mode of action lead to its functional diversity. This mitochondrial protease upon maturation, exposes its one-of-a-kind N-terminal tetrapeptide (AVPS) motif that binds and subsequently cleaves Inhibitor of Apoptosis Proteins (IAPs) thus promoting cell death, and posing as an important molecule for therapeutic intervention. Interestingly, unlike its other human counterparts, HtrA2 has also been implicated in maintaining the mitochondrial integrity through a bi-functional chaperone-protease activity, the *on-off* switch of which is yet to be identified. Furthermore, its ability to activate a wide repertoire of substrates through both its N- and C-terminal regions presumably has calibrated its association with several cellular pathways and hence diseases including neurodegenerative disorders and cancer. Therefore, the exclusive structural attributes of HtrA2 that involve multimodal activation, intermolecular PDZ-protease crosstalk, and an allosterically-modulated trimeric active-site ensemble have enabled the protease to evolve across species and partake functions that are fine-tuned for maintaining cellular homeostasis and mitochondrial proteome quality control in humans. These unique features along with its multitasking potential make HtrA2 a promising therapeutic target both in cancer and neurodegeneration.

## History and Background

The highly conserved high temperature requirement A (HtrA) family of serine proteases that perform a multitude of diverse physiological functions, constitute the core group of cellular proteases (Page and Di Cera, 2008). A complex oligomeric architecture (spanning from trimeric to 24-meric forms), which include an atypical N-terminal region, a conserved protease domain along with one or two C-terminal PDZ (postsynaptic density of 95 kDa, disc big, and zonula occludens 1) domains in each monomeric subunit make this family stand out among all other serine proteases (Clausen et al., 2002). Interestingly, the N-terminal regions of HtrAs exhibit significant sequence, size, and structural variability that encompass single transmembrane domain (prokaryotic DegS and human HtrA2), signal sequences, insulin-like growth factor-binding domains, and serine protease inhibitor domains (human HtrA1, HtrA3, and HtrA4) implicating intra- and inter-species functional divergence. Furthermore, their catalytic activity that can be allosterically tuned through an intricate rheostatic *on/off* switch as well as the modulatory protein-protein interaction domain(s) *aka* PDZ, has garnered much attention for their immense translational possibilities.

Interestingly, unlike eukaryotes and bacteria, archaean genomes are devoid of HtrA homologs (Koonin and Aravind, 2002). Although, all sequenced Nematoda genomes including the model organism *Caenorhabditis elegans* lack HtrA-like genes, they do encode PDZ-containing proteins (Koonin and Aravind, 2002) thus underscoring the functional relevance of this regulatory domain in various cellular pathways. While bacterial HtrAs have been demonstrated to be involved in protein quality control processes such as protein folding, stress response, and degradation of misfolded cell envelope proteins (Clausen et al., 2002), this function is manifested in their mammalian counterparts through the elimination of misfolded proteins including growth factors, regulation of cell proliferation, migration and apoptosis (Grau et al., 2005; Hou et al., 2005; Kapri-Pardes et al., 2007; Moisoi et al., 2009).

**FIGURE 1:**
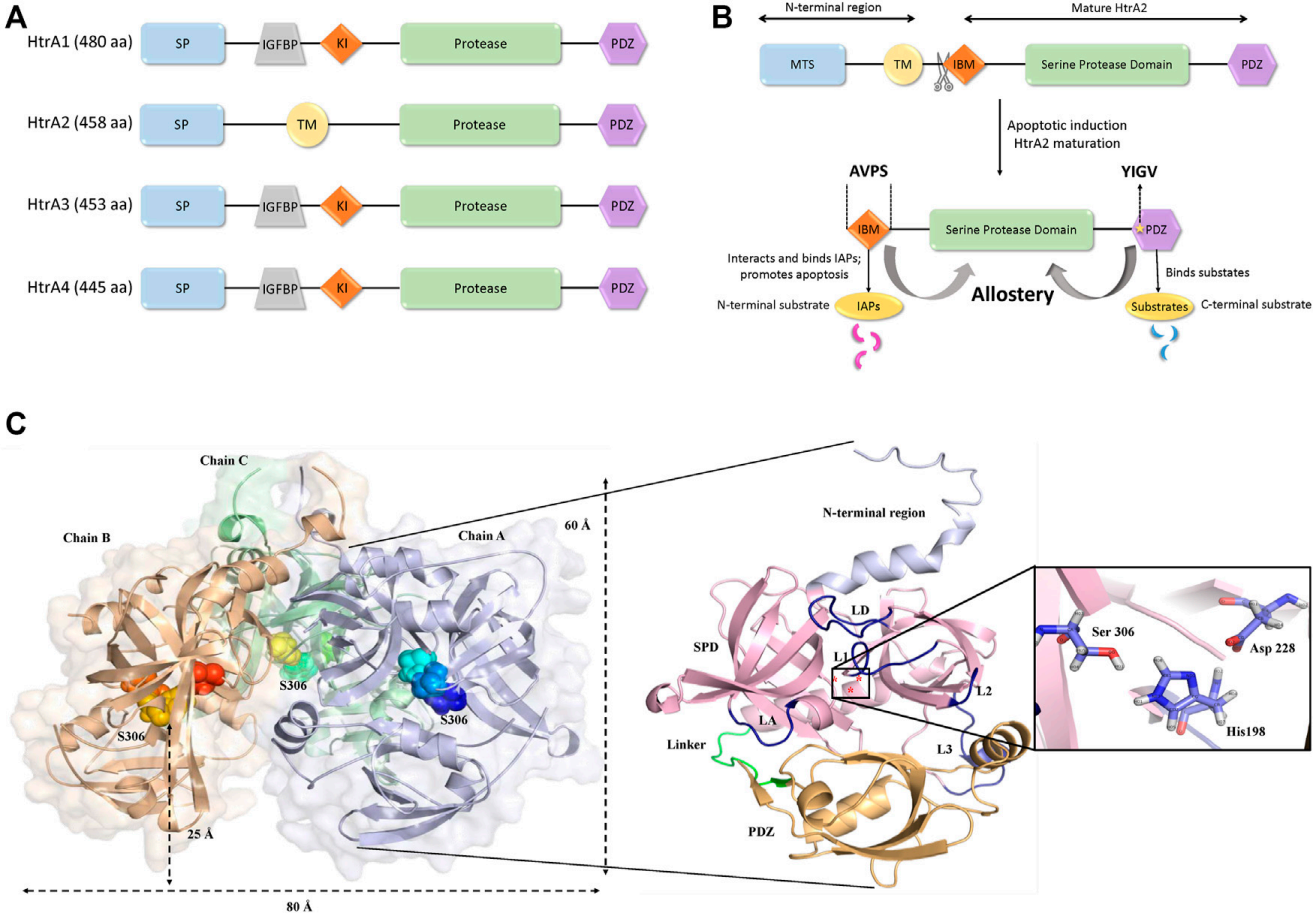
Structural organization of HtrA2. **(A)** Comparison of domain organizations of the human HtrA family including HtrA1, HtrA2, HtrA3 andHtrA4. aa, amino acid; SP, signal peptide; IGFBP, insulin growth factor binding domain; KI, Kazal-type S protease inhibitor domain; protease, protease domain; PDZ, PDZ domain; TM, transmembrane regulatory domain. **(B)** Schematic representation of the full-length HtrA2 protein (1–458 aa) and its different components as shown in the figure. Upon apoptotic trigger, the mitochondrial localization signal (133 residues) from the N-terminus gets cleaved exposing a tetrapeptide IAP-binding motif (IBM/AVPS), and concomitantly releasing the protease into the cytosol. Subsequent substrate binding at N- and/or C-termini leads to allosteric protease activation and substrate cleavage as described in the text. **(C)** The three-dimensional trimeric model adopted from the crystal structure (PDB ID: 1LCY) of HtrA2 highlighting the hidden catalytic triad (rainbow spheres) 25 Å above base of the pyramid (left side) while a single monomer has been zoomed into for describing the loops (yellow) and domains (N-terminal region: light purple, SPD: pink, PDZ: orange); the catalytic site has been shown in the inset (right side). L1, L2, L3 and LD are loops; SPD is serine protease domain and Linker represents the flexible region between the SPD and PDZ domain.

Among the four human HtrAs (HtrA1-4) that have been identified to date, HtrA2 has been most widely studied due to its enigmatic structural characteristics and profound functional relevance. While HtrA2 is found in the mitochondrial intermembrane space (IMS), its paralogs HtrA1, 3, and 4 are mostly found in the secretory process. Despite similar overall structural signature and conserved protease and PDZ domain architecture, these enzymes show a significant divergence in their N-terminal regions that might be essential for catering to their distinct functional properties For example, the N-terminal regions of HtrA1, 3, and 4 include secretory signals, along with insulin-like growth factor binding motifs and Kazal-type S protease inhibitor domains, while HtrA2 contains a mitochondrial localization signal (Figures 1A,B).

HtrA2, with a pyramid-shaped trimeric ensemble, is unique among its peers being the only known mitochondrial protease with a PDZ domain that identifies exposed hydrophobic regions of misfolded proteins (Li et al., 2002; Clausen et al., 2011; Singh et al., 2011). Furthermore, with the triggering of apoptotic signal, mature HtrA2 gets released from the mitochondrial IMS into the cytosol at the expense of its first 133 amino acid residues (Figure 1B). This series of events exposes an N-terminal tetrapeptide motif (AVPS) that binds to the Inhibitor of Apoptosis Proteins (IAPs) and abate their inhibition on caspases thus promoting apoptosis. Furthermore, HtrA2 is known to participate in apoptosis through both caspase-dependent and independent pathways, the latter through its serine protease activity (Hegde et al., 2002; Martins et al., 2002; Verhagen et al., 2002). Apart from its prominent role as a proapoptotic molecule, its involvement in neurodegenerative disorders has also been established through a missense mutation (Ser276Cys) in transgenic mice that exhibited motor neuron degeneration 2 (*mnd*2) implicating a Parkinsonian phenotype in humans (Jones et al., 2003). Further functional and clinical studies established HtrA2’s involvement in several neurodegenerative disorders (Inagaki et al., 2008; Kang et al., 2013; Wagh and Bose, 2018; Bose et al., 2021).

## Structural Features of HtrA2

Several efforts over the past decade have been made to capture the structural complexity of this proapoptotic enzyme from various perspectives. Shi and co-workers first provided the snapshots of the inactive (S306A) substrate-unbound form of mature HtrA2 in three-dimensional space (Li et al., 2002). The structural data showcased a trimeric pyramidal architecture with the short N-terminal regions upholding the oligomeric ensemble through van der Waals interactions, while three PDZ domains at the base encapsulated the active-sites of the protease domains. The protease domain that embeds a hydrophobic active-site pocket with the catalytic triad (Ser306, His198, and Asp228) forms a compact structural fold comprising seven α-helices and 19 β-strands. Surrounded by several regulatory and specificity loops, this domain is positioned deep within the oligomer at 25 Å above the base of the pyramid (Figure 1C) suggesting the requirement of substantial conformational changes for substrate binding and subsequent cleavage. The core of the pyramid is flanked by the regulatory PDZ domains that recognize and bind to the C-terminal region of their interacting partners. This is achieved through the canonical PDZ binding groove (YIGV) that is integrated into the PDZ-protease domain interface. The structural study also demonstrates that several non-covalent interactions in the substrate-unbound state keep the protease domain in its ‘*closed*’ conformation, through inhibitory interference from the surrounding PDZ domains.

Although, this structure provided an excellent overview of the HtrA2 structure, this substrate-unbound form of the protease failed to explain the underlying dynamics of its mode of activation. Most importantly, the model’s inability to enumerate the necessity to have a trimeric structure for its enzymatic functions as well as the mode of its distal allosteric regulation, impelled scientists to unravel the minutiae of its interactions from a more physiological as well as quantitative perspectives.

## Active Site Conformation and Multiple Activation Mechanisms of HtrA2

The pre-defined conserved domains of HtrA2, along with its regulatory (L1, L3, and LD) and specificity (L3-that accommodates specificity pocket) loops contribute to the activation mechanism of HtrA2 through multiple regulatory nodes (Figure 1C). Since these dynamic loops were mostly unresolved in the crystal structure, several efforts were made to investigate the multimodal allosteric regulation of the protease as well as understand the intricacies of HtrA2-mediated substrate cleavage (Martins et al., 2003; Jarzab et al., 2016). Because the allosteric binding partners are also predominantly its substrates (such as IAPS, GRIM-19, and Dusp-9), therefore the stepwise concerted allosteric mechanism either individually or in collaboration with different activation pathways could not be unequivocally determined using discrete peptide libraries. To circumvent the problem, Bose and co-workers utilized enzymology and biophysical approaches to understand the intricate coordination between the protease domain and other regions of the protein using full-length binding partners and/or substrates. Using β-casein, the generic substrate of serine proteases, Chaganti *et al.*, revisited the pre-existing model of HtrA2 activation and propounded a new hypothesis that relies on inter-molecular protease-PDZ crosstalk for initial substrate binding at the PDZ domain and its subsequent cleavage (Chaganti et al., 2013). This study identified interaction between the PDZ domain of one monomer with the serine protease domain of an adjacent one, which led to the rearrangement of H65 of the catalytic triad in a way to form a proper oxyanion hole. This series of inter-molecular making and breaking of bonds unequivocally demonstrated the requirement of the trimeric architecture for its allosteric propagation and activation by capturing the dynamics of the PDZ- and temperature-mediated activation process. Singh *et al.*, built upon the previous studies on N-terminal mediated activation of HtrA2 (Verhagen et al., 2002) and described the global conformational plasticity and subtle conformational reorientations in the loop regions surrounding the active-site to be involved in this process. Interestingly, using quantitative enzyme kinetics studies, they further demonstrated that the N-terminal mediated activation might also be regulated by PDZ-bound allosteric modulators and *vice-versa* (Singh et al., 2011; Singh et al., 2014) to bring the protease to the most competent catalytic state.

**FIGURE 2:**
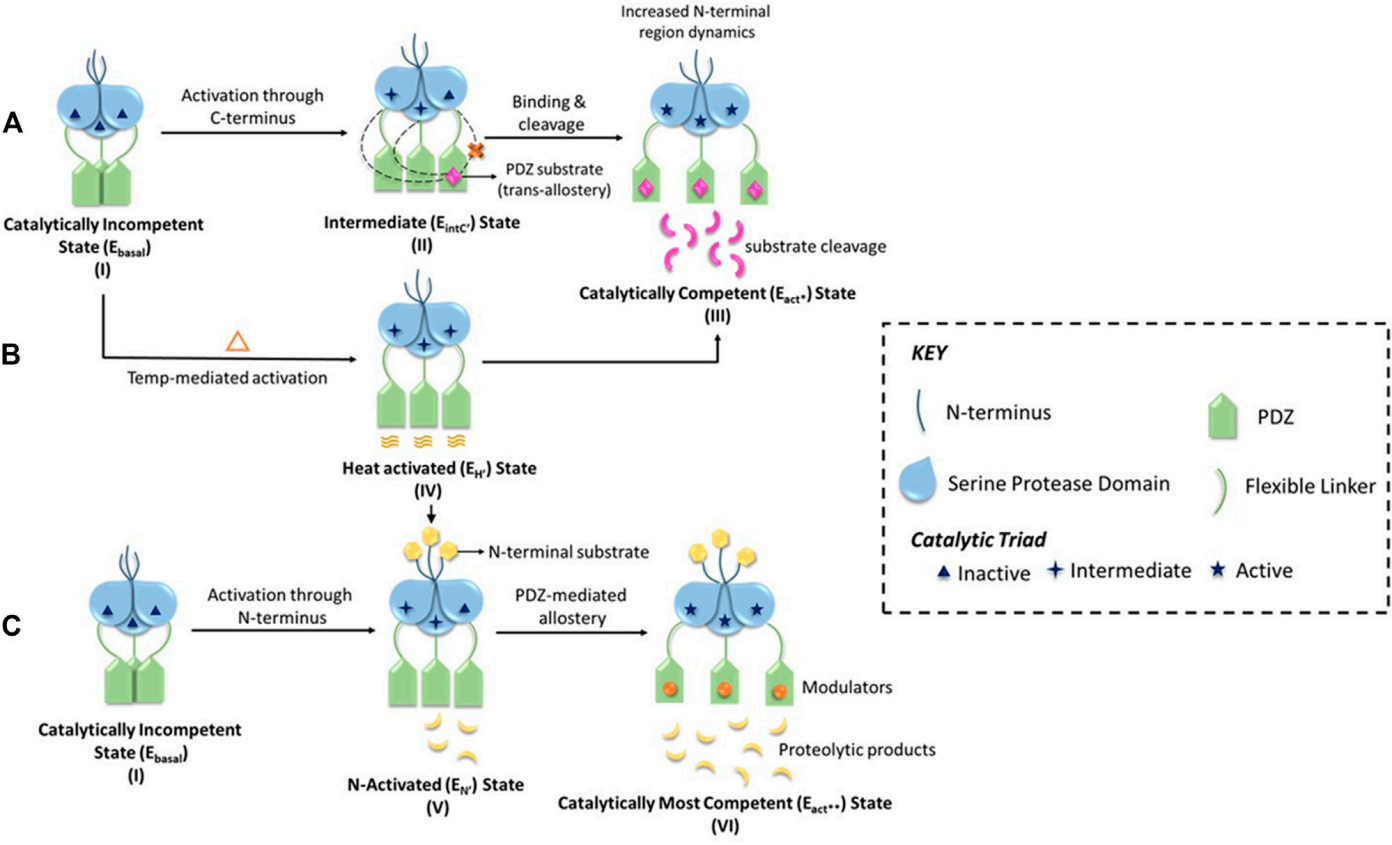
A schematic representation of the multimodal activation mechanism of HtrA2. **(A)** HtrA2 in the substrate-unbound state **(I)** exhibits negligible activity (*E*_*basal*_ *state*) since the active site it embedded within the trimeric ensemble, and encircled by the regulatory PDZ domains. In the second step **(II)**, initial binding of one substrate molecule at the distal PDZ domain (e.g., DUSP-9) reorients different domains and loops of HtrA2 making it conducive for catalysis (*E*_*IntC’*_^*’*^ *state or C-terminal mediated intermediate state*). In step **(II)**, the dotted lines from PDZ toward adjacent SPD domains highlight intermolecular crosstalk, which is shown to be absent within the same molecule through a red ‘x’ sign. Therefore, this step underscores the importance of *trans*-mediated allostery where binding of substrate molecule to a PDZ activates protease domains of the adjacent subunits of the trimer. Further binding of substrates to all the PDZ domains **(III)**, activates the protease via conformational alterations throughout the protein molecule leading to an optimally active state (*E*_act*_ *state*). **(B)** On the other hand, with increase in temperature, the PDZs experience enhanced thermal motion leading to an open active-site conformation (*E*_*H*_ *’ state or heat-mediated active state*) as shown in **(IV)**. This conformation might be important in some scenarios for preparing the basal protease to readily bind substrates of distinct cellular pathways either/both at the N-terminal or/and the PDZ domains (shown by arrows). **(C)** Represents N-terminal mediated allosteric activation of HtrA2, where the basal state **(I)** binds to the N-terminal binding partners (such as IAPs) of HtrA2 that leads to favorable conformational alterations in the distal protease and PDZ domains leading to an active state (*E*_*N*_ *’ state or N-terminal mediated activated state*) as shown in step **(V)**. This conformational state can further be modulated through temperature and/or PDZ domains leading to the most active protease (*E*_act**_ *state*) as shown in **(VI)**. This unique model shows intricate crosstalk among distinct activation networks that might or might not be mutually exclusive depending upon specific cellular requirements.

Although these studies provided a holistic understanding of HtrA’s mode of activation through three distinct yet non-exclusive modes, they did not provide the stoichiometric contribution of the PDZ-protease communication in a step-by-step manner. Using molecular dynamics, protein engineering, structural and chemical biology approaches, two different groups (Parui et al., 2021; Toyama et al., 2021) distinctly established the *trans*-mediated PDZ-protease collaboration that espouses a unique reciprocative mechanism where the distal PDZ reorients the active site of the adjacent monomer and attunes it for catalysis through a precise synergistic relay of information. This multi-tiered regulation of HtrA2 activation might be critical toward prevention of untimely proteolysis as well as accurately controlling its involvement in different pathophysiological pathways such as apoptosis, protein quality-control, cancer, arthritis, and neurodegeneration, where it cleaves a wide spectrum of substrates in different subcellular locations. This is substantiated by the identification and characterization of protein-protein interactions involving HtrA2 and its substrates such as Inhibitor of Apoptosis Proteins (IAPs), hematopoietic cell-specific protein-1-(HS1)-associated protein X-1 (Hax-1), Dual-specificity phosphatase-9 (DUSP-9), a gene associated with retinoic and interferon-induced mortality-19 protein (GRIM-19) and Phosphoprotein enriched in astrocytes-15 (Pea-15) (Chaganti et al., 2019; Acharya et al., 2020; Kummari et al., 2021) that unlike other HtrAs are interestingly not restricted to the C-terminal PDZ domains. The holistic enumeration of HtrA2’s activation network has been vividly illustrated in Figure 2 and the mechanism is elaborated in the figure legend.

This chain of ground-breaking revelations on the reciprocity of its structural dynamism and multifarious physiological as well as disease-associated functions as discussed below have opened up avenues to regulate HtrA2 functions at various check-points toward devising customized therapeutic strategies.

## Is HtrA2 a Chaperone?

The neurodegenerative phenotype of mice lacking HtrA2 or harboring the enzymatically inactive *mnd2*mutant (S276C) implies that HtrA2 protease activity protects neuronal mitochondria (Jones et al., 2003; Martins et al., 2004). It was earlier speculated that HtrA2 monitors and regulates protein folding in the mitochondria in a way DegP does in the bacterial periplasm. Further studies demonstrated that unfolded protein response (UPR) induced by tunicamycin or heat shock (Gray et al., 2000) as well as etoposide-activatedp53 stress pathway-upregulated expression of HtrA2 protease (Jin et al., 2003). Alike DegP, HtrA2 is also activated by elevated temperatures (Martins et al., 2003). Moreover, both HtrA2 and DegP prefer aliphatic Val or Ile in P1 position for substrate recognition and cleavage (Kolmar et al., 1996). Despite these similarities, HtrA2 shares strikingly higher structural and functional traits with DegS, which argue against its DegP-like chaperoning function, and hints a bearing closer to DegS. In particular, HtrA2 is protease-active at room temperature (Savopoulos et al., 2000), while DegP is activated only at elevated temperatures (Spiess et al., 1999). In addition, while DegP with two PDZ domains, folds into a higher-order hexagonal cage (Krojer et al., 2002), the trimeric HtrA2 and DegS (sans the additional PDZ and the necessary longer LA loop) are unable to prevent the entry of correctly folded proteins into the proteolytic sites (Clausen et al., 2002; Kim et al., 2003; Kim and Kim, 2005; Clausen et al., 2011) thus creating certain equivocacies toward defining its role as a chaperone. Interestingly, the identification of presenilin and amyloid precursor protein as natural substrates of HtrA2 (Gray et al., 2000; Gupta et al., 2004) necessitates further studies to resolve the ambiguities surrounding HtrA’s role in unfolded protein aggregation and quality control.

## Role of HtrA2 in Apoptosis

HtrA2 was the first to be identified as an IAP binding protein (Hegde et al., 2002). Its functional similarity with second mitochondria-derived activator of caspase (Smac)/direct IAP binding protein with low pI (DIABLO) established its role as a proapoptotic molecule (Martins et al., 2002; Suzuki et al., 2004). HtrA2, which resides in the mitochondrial IMS is released into the cytosol after the separation of its 133-residue mitochondrial localization signal. This exposes an N-terminal IAP-binding motif (IBM) comprising a tetrapeptide ‘AVPS’ that is recognized as a binding site for IAPs. Unlike Smac, HtrA2 also cleaves IAPs and hence irrevocably relieves their inhibition on caspases (caspases-3,-7, and -9), thus promoting apoptosis (Srinivasula et al., 2003; Yang et al., 2003). Conservation of the IBM motif is found across species, where its *Drosophila* ortholog with two IBM motifs attracts DIAP1, enabling its removal by the serine protease activity (Challa et al., 2007). Likewise, the rhesus monkey and rodent orthologs of the protease have maintained the IBM motif suggesting evolutionary diversification of HtrA2 functions in higher organisms (Vande Walle et al., 2008). Interestingly, the two IAP-related proteins in *C. elegans* do not appear to be involved in apoptosis regulation (Fraser et al., 1999; Speliotes et al., 2000), suggesting that IAP proteins and the appearance of IAP antagonists like HtrA2 and *Drosophila* Reaper, Hid, and Grim are recent additions to the apoptotic molecular repertoire. Although human HtrA2 and its evolutionary paralogs bind and degrade many IAP family members, XIAP is found as the most effective amongst them as it engages a second interaction surface that permits strong caspase inhibition (Eckelman et al., 2006). However, to inhibit caspase activation, cIAP1,cIAP2, and XIAP target bound caspases for ubiquitin-mediated proteasomal degradation (Vaux and Silke, 2005) thus necessitating HtrA2 to cleave all of them.

Apart from N-terminal mediated apoptosis, HtrA2 binds important molecules of the apoptotic pathway through its regulatory C-terminal PDZ domain. The binding of substrates to the hydrophobic YIGV groove allosterically activates the protease for substrate binding and subsequent catalysis. Furthermore, binding to mitochondrial substrates at the early apoptotic stage such as GRIM-19 and Hax-1 might be important toward attuning the mature protease for its proapoptotic functions before it enters the cytoplasm (Cilenti et al., 2004; Ma et al., 2007; Chaganti et al., 2019; Kummari et al., 2021) where it binds several antiapoptotic proteins including IAPs and death effector domain (DED) containing Pea-15 (Trencia et al., 2004). HtrA2 is also capable of inducing caspase-independent apoptosis via its serine protease activity by cleaving several critical cellular molecules such as cytoskeletal proteins (actin, α-/β-tubulin, and vimentin) that are important for upholding cellular integrity (Vande Walle et al., 2007). KIAA1967 and KIAA0251are two newly identified proteins of the apoptotic pathway that have been found to be substrates of HtrA2 (Vande Walle et al., 2007). A caspase-generated cleavage fragment of KIAA1967 was demonstrated to cause mitochondrial clustering and matrix condensation in apoptotic *HeLa* cells (Sundararajan et al., 2005), whereas KIAA0251 interacts with the endoplasmic reticulum (ER) membrane protein Bap29, a component known to be required for caspase-8 activation in the ER (Breckenridge et al., 2002). Taken together, the substrates found and verified for HtrA2, reveal that this protease is involved in the apoptotic process at the cytoskeleton, translation initiation complex, and organelle dismantling levels.

Multiple modes of activation and a variety of substrates in different subcellular locations make HtrA2 omnipresent in the apoptotic pathway. Furthermore, distal N-/C-termini and heat-mediated positive allosteric modulation as well as negative regulation of its proapoptotic functions through phosphorylation at Ser212(Yang et al., 2016) re-instate its enigmatic role in the cell death network. However, the lack of definitive *in vivo* models of HtrA2’s contribution toward apoptotic pathway might be limited by the number of identified natural substrates to date as well as due to redundancy in its functions in the cell, which requires further investigations.

## HtrA2 in Neurodegenerative Disorders and Cancer

The first report on HtrA2’s involvement in neurodegeneration came into existence with the identification of its interaction with Alzheimer’s disease-associated protein, presenilin-1 (Gupta et al., 2004). This was later substantiated by a homozygous *loss-of-function* mutation (S276C) identified as motor neurodegeneration 2 (*mnd*2) in mice (Jones et al., 2003), which was further bolstered by the development of homozygous *HTRA2* knock-out mice exhibiting Parkinsonian phenotype (Martins et al., 2004) thus assigning *HTRA2* gene the PARK13(Parkinson’s disease 13) locus (Strauss et al., 2005; Abou-Sleiman et al., 2006). These critical inputs led to the initiation of several clinical studies involving PD cohorts from various populations across the globe to identify the involvement of *HTRA2* and its mutations in PD progression and pathogenesis. However, the data obtained were quite contrasting. For example, a Germany-based clinical study that demonstrated heterozygous G399S and A141S mutations (Strauss et al., 2005; Bogaerts et al., 2008), was later impugned by another study from North America (Simon-Sanchez and Singleton, 2008). However, *in vivo* studies in transgenic mice harboring the G399S mutation (Casadei et al., 2016) and several other independent clinical investigations on non-overlapping rare *HTRA2* mutations in Asian and European populations, re-established the correlation between *HTRA2* gene and PD risk (Bogaerts et al., 2008; Lin et al., 2011; Wang et al., 2011). Furthermore, to delve into the loss of enzymatic activity of S276C mutation in human HtrA2 and correlate it with PD if any, X-ray crystallographic studies of the mutant were performed to understand the structural correlates of this functional repercussion (Wagh and Bose, 2018). The study provided a structural snapshot of the mutant at an atomic resolution where the inactivity was found to be conferred by loss of water-mediated H-bond between residues S276 and I270 on regulatory L2 and LD loops respectively; however, no clinical study could identify S276C mutation in any PD patient. Recently, another patient-derived research in the Indian population identified a rare likely-pathogenic mutation (T242M), which is critical for altering mitochondrial homeostasis due to loss of GSK-3β-mediated phosphorylation on HtrA2 leading to uncontrolled cell death with PD phenotype (Bose et al., 2021). Moreover, another contemporary study demonstrates a connection between neuronal death and selective downregulation revealing its link with Huntington’s disease (Inagaki et al., 2008).

Despite these crucial discoveries, several contradictory reports challenge the establishment of HtrA2’s role in neurodegeneration. This apparent anomaly in these studies might be due to a lack of focus on close interconnections among several parameters that include alterations in *HTRA2*, mitochondrial functional aberrations, and neurodegeneration. Therefore, future research endeavors encompassing both genetic and epigenetic interactions underlying the complex pathophysiological network of neurodegenerative disorders might provide a more comprehensive picture of *HTRA2*’s association with these diseases.

While the involvement of HtrA1 in cancer is quite prevalent, there have been only a few direct reports of HtrA2’s association with oncogenesis. HtrA2 has been found to be widely expressed in several cancer cell lines where over-expression triggered cell death (Suzuki et al., 2001; Martins et al., 2002). Biopsy sample analyses of specific cancers exhibited altered expression of HtrA2 suggesting its role in those cancers. For example, the level of the protease was found to be substantially less in endometrial and ovarian cancer tissues (Narkiewicz et al., 2008; Narkiewicz et al., 2009). On the other hand, higher HtrA2 expression in prostate tumors implicated its association with the differentiation of prostate cancer cells (Hu et al., 2006). Furthermore, elevated levels of HtrA2 in gastric cancers link it with this malignancy (Lee et al., 2003). However, although, the contribution of HtrA2 toward cancer development or regression yet remains to be conclusively elucidated, future studies using multidisciplinary approaches for delineating the HtrA2-associated extensive apoptotic network, and identifying its effect on tumorigenesis might shed more light on this pathophysiological collaboration.

## Concluding Remarks and Future Perspective

Recent progress in the structural and functional characterization of HtrA2 has greatly enhanced our understanding of this fascinating protein. Association of this protease with critical cellular functions such as apoptosis, protein quality control, cell growth, and unfolded protein response implicate it in several diseases including neurodegeneration, arthritis, and cancer. Unfortunately, the complexity of its oligomeric structural constitution and mechanism of activation makes it one of the most complex molecules in the HtrA family of proteases. However, recent advancements in deciphering the multi-layered allosteric modulation of HtrA2 from both structural and functional perspectives provide important cues toward targeting its different functions with specific modulators having desired characteristics.

## References

[B1] Abou-Sleiman P. M. Muqit M. M. K. Wood N. W. (2006). Expanding Insights of Mitochondrial Dysfunction in Parkinson's Disease. Nat. Rev. Neurosci. 7 (3), 207–219. 10.1038/nrn1868 16495942

[B2] Acharya S. Dutta S. Mudrale S. P. Bose K. (2020). Dual Specificity Phosphatase 9: A Novel Binding Partner Cum Substrate of Proapoptotic Serine Protease HtrA2. Biochem. Biophysical Res. Commun. 533 (3), 607–612. 10.1016/j.bbrc.2020.09.062 32988583

[B3] Bogaerts V. Nuytemans K. Reumers J. Pals P. Engelborghs S. Pickut B. (2008). Genetic Variability in the Mitochondrial Serine proteaseHTRA2contributes to Risk for Parkinson Disease. Hum. Mutat. 29 (6), 832–840. 10.1002/humu.20713 18401856

[B4] Bose K. Wagh A. Mishra V. Dutta S. Parui A. L. Puja R. (2021). Loss of GSK-3β Mediated Phosphorylation in HtrA2 Contributes to Uncontrolled Cell Death with Parkinsonian Phenotype. Int. J. Biol. Macromolecules 180, 97–111. 10.1016/j.ijbiomac.2021.03.040 33716130

[B5] Breckenridge D. G. Nguyen M. Kuppig S. Reth M. Shore G. C. (2002). The Procaspase-8 Isoform, procaspase-8L, Recruited to the BAP31 Complex at the Endoplasmic Reticulum. Proc. Natl. Acad. Sci. 99 (7), 4331–4336. 10.1073/pnas.072088099 11917123 PMC123648

[B6] Casadei N. Sood P. Ulrich T. Fallier-Becker P. Kieper N. Helling S. (2016). Mitochondrial Defects and Neurodegeneration in Mice Overexpressing Wild-type or G399S Mutant HtrA2. Hum. Mol. Genet. 25 (3), 459–471. 10.1093/hmg/ddv485 26604148

[B7] Chaganti L. K. Dutta S. Kuppili R. R. Mandal M. Bose K. (2019). Structural Modeling and Role of HAX-1 as a Positive Allosteric Modulator of Human Serine Protease HtrA2. Biochem. J. 476 (20), 2965–2980. 10.1042/BCJ20190569 31548268

[B8] Chaganti L. K. Kuppili R. R. Bose K. (2013). Intricate Structural Coordination and Domain Plasticity Regulate Activity of Serine Protease HtrA2. FASEB j. 27 (8), 3054–3066. 10.1096/fj.13-227256 23608143

[B9] Challa M. Malladi S. Pellock B. J. Dresnek D. Varadarajan S. Yin Y. W. (2007). Drosophila Omi, a Mitochondrial-Localized IAP Antagonist and Proapoptotic Serine Protease. EMBO J. 26 (13), 3144–3156. 10.1038/sj.emboj.7601745 17557079 PMC1914093

[B10] Cilenti L. Soundarapandian M. M. Kyriazis G. A. Stratico V. Singh S. Gupta S. (2004). Regulation of HAX-1 Anti-apoptotic Protein by Omi/HtrA2 Protease during Cell Death. J. Biol. Chem. 279 (48), 50295–50301. 10.1074/jbc.M406006200 15371414

[B11] Clausen T. Kaiser M. Huber R. Ehrmann M. (2011). HTRA Proteases: Regulated Proteolysis in Protein Quality Control. Nat. Rev. Mol. Cel Biol 12 (3), 152–162. 10.1038/nrm3065 21326199

[B12] Clausen T. Southan C. Ehrmann M. (2002). The HtrA Family of Proteases. Mol. Cel 10 (3), 443–455. 10.1016/s1097-2765(02)00658-5 12408815

[B13] Eckelman B. P. Salvesen G. S. Scott F. L. (2006). Human Inhibitor of Apoptosis Proteins: Why XIAP Is the Black Sheep of the Family. EMBO Rep. 7 (10), 988–994. 10.1038/sj.embor.7400795 17016456 PMC1618369

[B14] Fraser A. G. James C. Evan G. I. Hengartner M. O. (1999). *Caenorhabditis elegans* Inhibitor of Apoptosis Protein (IAP) Homologue BIR-1 Plays a Conserved Role in Cytokinesis. Curr. Biol. 9 (6), 292–302. 10.1016/s0960-9822(99)80137-7 10209096

[B15] Grau S. Baldi A. Bussani R. Tian X. Stefanescu R. Przybylski M. (2005). Implications of the Serine Protease HtrA1 in Amyloid Precursor Protein Processing. Proc. Natl. Acad. Sci. 102 (17), 6021–6026. 10.1073/pnas.0501823102 15855271 PMC1087941

[B16] Gray C. W. Ward R. V. Karran E. Turconi S. Rowles A. Viglienghi D. (2000). Characterization of Human HtrA2, a Novel Serine Protease Involved in the Mammalian Cellular Stress Response. Eur. J. Biochem. 267 (18), 5699–5710. 10.1046/j.1432-1327.2000.01589.x 10971580

[B17] Gupta S. Singh R. Datta P. Zhang Z. Orr C. Lu Z. (2004). The C-Terminal Tail of Presenilin Regulates Omi/HtrA2 Protease Activity. J. Biol. Chem. 279 (44), 45844–45854. 10.1074/jbc.M404940200 15294909

[B18] Hegde R. Srinivasula S. M. Zhang Z. Wassell R. Mukattash R. Cilenti L. (2002). Identification of Omi/HtrA2 as a Mitochondrial Apoptotic Serine Protease that Disrupts Inhibitor of Apoptosis Protein-Caspase Interaction. J. Biol. Chem. 277 (1), 432–438. 10.1074/jbc.M109721200 11606597

[B19] Hou J. Clemmons D. R. Smeekens S. (2005). Expression and Characterization of a Serine Protease that Preferentially Cleaves Insulin-like Growth Factor Binding Protein-5. J. Cel. Biochem. 94 (3), 470–484. 10.1002/jcb.20328 15534875

[B20] Hu X.-Y. Xu Y.-M. Chen X. C. Ping H. Chen Z.-H. Zeng F.-Q. (2006). Immunohistochemical Analysis of Omi/HtrA2 Expression in Prostate Cancer and Benign Prostatic Hyperplasia. APMIS 114 (12), 893–898. 10.1111/j.1600-0463.2006.apm_271.x 17207090

[B21] Inagaki R. Tagawa K. Qi M.-L. Enokido Y. Ito H. Tamura T. (2008). Omi/HtrA2 Is Relevant to the Selective Vulnerability of Striatal Neurons in Huntington's Disease. Eur. J. Neurosci. 28 (1), 30–40. 10.1111/j.1460-9568.2008.06323.x 18662332

[B22] Jarzab M. Wenta T. Zurawa-Janicka D. Polit A. Gieldon A. J. Wysocka M. (2016). Intra- and Intersubunit Changes Accompanying thermal Activation of the HtrA2(Omi) Protease Homotrimer. Biochim. Biophys. Acta (Bba) - Proteins Proteomics 1864 (3), 283–296. 10.1016/j.bbapap.2015.12.002 26702898

[B23] Jin S. Kalkum M. Overholtzer M. Stoffel A. Chait B. T. Levine A. J. (2003). CIAP1 and the Serine Protease HTRA2 Are Involved in a Novel P53-dependent Apoptosis Pathway in Mammals. Genes Dev. 17 (3), 359–367. 10.1101/gad.1047003 12569127 PMC195984

[B24] Jones J. M. Datta P. Srinivasula S. M. Ji W. Gupta S. Zhang Z. (2003). Loss of Omi Mitochondrial Protease Activity Causes the Neuromuscular Disorder of Mnd2 Mutant Mice. Nature 425 (6959), 721–727. 10.1038/nature02052 14534547

[B25] Kang S. Louboutin J.-P. Datta P. Landel C. P. Martinez D. Zervos A. S. (2013). Loss of HtrA2/Omi Activity in Non-neuronal Tissues of Adult Mice Causes Premature Aging. Cell Death Differ 20 (2), 259–269. 10.1038/cdd.2012.117 22976834 PMC3554338

[B26] Kapri-Pardes E. Naveh L. Adam Z. (2007). The Thylakoid Lumen Protease Deg1 Is Involved in the Repair of Photosystem II from Photoinhibition in Arabidopsis. Plant Cell 19 (3), 1039–1047. 10.1105/tpc.106.046573 17351117 PMC1867356

[B27] Kim D.-Y. Kim K.-K. (2005). Structure and Function of HtrA Family Proteins, the Key Players in Protein Quality Control. BMB Rep. 38 (3), 266–274. 10.5483/bmbrep.2005.38.3.266 15943900

[B28] Kim D. Y. Kim D. R. Ha S. C. Lokanath N. K. Lee C. J. Hwang H.-Y. (2003). Crystal Structure of the Protease Domain of a Heat-Shock Protein HtrA from Thermotoga Maritima. J. Biol. Chem. 278 (8), 6543–6551. 10.1074/jbc.M208148200 12458220

[B29] Kolmar H. Waller P. R. Sauer R. T. (1996). The DegP and DegQ Periplasmic Endoproteases of *Escherichia coli*: Specificity for Cleavage Sites and Substrate Conformation. J. Bacteriol. 178 (20), 5925–5929. 10.1128/jb.178.20.5925-5929.1996 8830688 PMC178448

[B30] Koonin E. V. Aravind L. (2002). Origin and Evolution of Eukaryotic Apoptosis: the Bacterial Connection. Cel Death Differ 9 (4), 394–404. 10.1038/sj.cdd.4400991 11965492

[B31] Krojer T. Garrido-Franco M. Huber R. Ehrmann M. Clausen T. (2002). Crystal Structure of DegP (HtrA) Reveals a New Protease-Chaperone Machine. Nature 416 (6879), 455–459. 10.1038/416455a 11919638

[B32] Kummari R. Dutta S. Patil S. Mudrale S. P. Bose K. (2021). Elucidating the Role of GRIM-19 as a Substrate and Allosteric Activator of Pro-apoptotic Serine Protease HtrA2. Biochem. J. 478 (6), 1241–1259. 10.1042/BCJ20200923 33650635

[B33] Lee S. H. Lee J. W. Kim H. S. Kim S. Y. Park W. S. Kim S. H. (2003). Immunohistochemical Analysis of Omi/HtrA2 Expression in Stomach Cancer. APMIS 111 (5), 586–590. 10.1034/j.1600-0463.2003.1110508.x 12887511

[B34] Li W. Srinivasula S. M. Chai J. Li P. Wu J.-W. Zhang Z. (2002). Structural Insights into the Pro-apoptotic Function of Mitochondrial Serine Protease HtrA2/Omi. Nat. Struct. Biol. 9 (6), 436–441. 10.1038/nsb795 11967569

[B35] Lin C.-H. Chen M.-L. Chen G. S. Tai C.-H. Wu R.-M. (2011). Novel Variant Pro143Ala in HTRA2 Contributes to Parkinson's Disease by Inducing Hyperphosphorylation of HTRA2 Protein in Mitochondria. Hum. Genet. 130 (6), 817–827. 10.1007/s00439-011-1041-6 21701785 PMC3214265

[B36] Lipinska J. Lapinska-Szumczyk S. Zurawa-Janicka D. Skorko-Glonek J. Emerich J. Lipinska B. (2009). Expression of Human HtrA1, HtrA2, HtrA3 and TGF-Β1 Genes in Primary Endometrial Cancer. Oncol. Rep. 21 (6), 1529–1537. 10.3892/or_00000385 19424634

[B37] Ma X. Kalakonda S. Srinivasula S. M. Reddy S. P. Platanias L. C. Kalvakolanu D. V. (2007). GRIM-19 Associates with the Serine Protease HtrA2 for Promoting Cell Death. Oncogene 26 (33), 4842–4849. 10.1038/sj.onc.1210287 17297443

[B38] Martins L. M. Iaccarino I. Tenev T. Gschmeissner S. Totty N. F. Lemoine N. R. (2002). The Serine Protease Omi/HtrA2 Regulates Apoptosis by Binding XIAP through a Reaper-like Motif. J. Biol. Chem. 277 (1), 439–444. 10.1074/jbc.M109784200 11602612

[B39] Martins L. M. Morrison A. Klupsch K. Fedele V. Moisoi N. Teismann P. (2004). Neuroprotective Role of the Reaper-Related Serine Protease HtrA2/Omi Revealed by Targeted Deletion in Mice. Mol. Cel Biol 24 (22), 9848–9862. 10.1128/MCB.24.22.9848-9862.2004 PMC525490 15509788

[B40] Martins L. M. Turk B. E. Cowling V. Borg A. Jarrell E. T. Cantley L. C. (2003). Binding Specificity and Regulation of the Serine Protease and PDZ Domains of HtrA2/Omi. J. Biol. Chem. 278 (49), 49417–49427. 10.1074/jbc.M308659200 14512424

[B41] Moisoi N. Klupsch K. Fedele V. East P. Sharma S. Renton A. (2009). Mitochondrial Dysfunction Triggered by Loss of HtrA2 Results in the Activation of a Brain-specific Transcriptional Stress Response. Cel Death Differ 16 (3), 449–464. 10.1038/cdd.2008.166 19023330

[B42] Narkiewicz J. Klasa-Mazurkiewicz D. Zurawa-Janicka D. Skorko-Glonek J. Emerich J. Lipinska B. (2008). Changes in mRNA and Protein Levels of Human HtrA1, HtrA2 and HtrA3 in Ovarian Cancer. Clin. Biochem. 41 (7-8), 561–569. 10.1016/j.clinbiochem.2008.01.004 18241672

[B43] Page M. J. Di Cera E. (2008). Evolution of Peptidase Diversity. J. Biol. Chem. 283 (44), 30010–30014. 10.1074/jbc.M804650200 18768474 PMC2573091

[B44] Parui A. L. Mishra V. Dutta S. Bhaumik P. Bose K. (2021). Inter-subunit Crosstalk via PDZ Synergistically Governs Allosteric Activation of Proapoptotic HtrA2. bioRxiv, 2021.2010.2004 2021, 462974. 10.1101/2021.10.04.462974 35738282

[B45] Savopoulos J. W. Carter P. S. Turconi S. Pettman G. R. Karran E. H. Gray C. W. (2000). Expression, Purification, and Functional Analysis of the Human Serine Protease HtrA2. Protein Expr. Purif. 19 (2), 227–234. 10.1006/prep.2000.1240 10873535

[B46] Simon-Sanchez J. Singleton A. B. (2008). Sequencing Analysis of OMI/HTRA2 Shows Previously Reported Pathogenic Mutations in Neurologically normal Controls. Hum. Mol. Genet. 17 (13), 1988–1993. 10.1093/hmg/ddn096 18364387 PMC2574854

[B47] Singh N. D'Souza A. Cholleti A. Sastry G. M. Bose K. (2014). Dual Regulatory Switch Confers Tighter Control on HtrA2 Proteolytic Activity. FEBS J. 281 (10), 2456–2470. 10.1111/febs.12799 24698088

[B48] Singh N. Kuppili R. R. Bose K. (2011). The Structural Basis of Mode of Activation and Functional Diversity: a Case Study with HtrA Family of Serine Proteases. Arch. Biochem. Biophys. 516 (2), 85–96. 10.1016/j.abb.2011.10.007 22027029

[B49] Speliotes E. K. Uren A. Vaux D. Horvitz H. R. (2000). The Survivin-like *C. elegans* BIR-1 Protein Acts with the Aurora-like Kinase AIR-2 to Affect Chromosomes and the Spindle Midzone. Mol. Cel 6 (2), 211–223. 10.1016/s1097-2765(00)00023-x 10983970

[B50] Spiess C. Beil A. Ehrmann M. (1999). A Temperature-dependent Switch from Chaperone to Protease in a Widely Conserved Heat Shock Protein. Cell 97 (3), 339–347. 10.1016/s0092-8674(00)80743-6 10319814

[B51] Srinivasula S. M. Gupta S. Datta P. Zhang Z. Hegde R. Cheong N. (2003). Inhibitor of Apoptosis Proteins Are Substrates for the Mitochondrial Serine Protease Omi/HtrA2. J. Biol. Chem. 278 (34), 31469–31472. 10.1074/jbc.C300240200 12835328

[B52] Strauss K. M. Martins L. M. Plun-Favreau H. Marx F. P. Kautzmann S. Berg D. (2005). Loss of Function Mutations in the Gene Encoding Omi/HtrA2 in Parkinson's Disease. Hum. Mol. Genet. 14 (15), 2099–2111. 10.1093/hmg/ddi215 15961413

[B53] Sundararajan R. Chen G. Mukherjee C. White E. (2005). Caspase-dependent Processing Activates the Proapoptotic Activity of Deleted in Breast Cancer-1 during Tumor Necrosis Factor-Alpha-Mediated Death Signaling. Oncogene 24 (31), 4908–4920. 10.1038/sj.onc.1208681 15824730

[B54] Suzuki Y. Imai Y. Nakayama H. Takahashi K. Takio K. Takahashi R. (2001). A Serine Protease, HtrA2, Is Released from the Mitochondria and Interacts with XIAP, Inducing Cell Death. Mol. Cel 8 (3), 613–621. 10.1016/s1097-2765(01)00341-0 11583623

[B55] Suzuki Y. Takahashi-Niki K. Akagi T. Hashikawa T. Takahashi R. (2004). Mitochondrial Protease Omi/HtrA2 Enhances Caspase Activation through Multiple Pathways. Cel Death Differ 11 (2), 208–216. 10.1038/sj.cdd.4401343 14605674

[B56] Toyama Y. Harkness R. W. Lee T. Y. T. Maynes J. T. Kay L. E. (2021). Oligomeric Assembly Regulating Mitochondrial HtrA2 Function as Examined by Methyl-TROSY NMR. Proc. Natl. Acad. Sci. USA 118 (11), e2025022118. 10.1073/pnas.2025022118 33692127 PMC7980377

[B57] Trencia A. Fiory F. Maitan M. A. Vito P. Barbagallo A. P. M. Perfetti A. (2004). Omi/HtrA2 Promotes Cell Death by Binding and Degrading the Anti-apoptotic Protein Ped/pea-15. J. Biol. Chem. 279 (45), 46566–46572. 10.1074/jbc.M406317200 15328349

[B58] Vande Walle L. Lamkanfi M. Vandenabeele P. (2008). The Mitochondrial Serine Protease HtrA2/Omi: an Overview. Cel Death Differ 15 (3), 453–460. 10.1038/sj.cdd.4402291 18174901

[B59] Vande Walle L. Van Damme P. Lamkanfi M. Saelens X. Vandekerckhove J. Gevaert K. (2007). Proteome-wide Identification of HtrA2/Omi Substrates. J. Proteome Res. 6 (3), 1006–1015. 10.1021/pr060510d 17266347

[B60] Vaux D. L. Silke J. (2005). IAPs - the Ubiquitin Connection. Cel Death Differ 12 (9), 1205–1207. 10.1038/sj.cdd.4401696 16094398

[B61] Verhagen A. M. Silke J. Ekert P. G. Pakusch M. Kaufmann H. Connolly L. M. (2002). HtrA2 Promotes Cell Death through its Serine Protease Activity and its Ability to Antagonize Inhibitor of Apoptosis Proteins. J. Biol. Chem. 277 (1), 445–454. 10.1074/jbc.M109891200 11604410

[B62] Wagh A. R. Bose K. (2018). Structural Basis of Inactivation of Human Counterpart of Mouse Motor Neuron Degeneration 2 Mutant in Serine Protease HtrA2. Biosci. Rep. 38 (5), BSR20181072. 10.1042/BSR20181072 30068699 PMC6172425

[B63] Wang C.-y. Xu Q. Weng L. Zhang Q. Zhang H.-n. Guo J.-f. (2011). Genetic Variations of Omi/HTRA2 in Chinese Patients with Parkinson's Disease. Brain Res. 1385, 293–297. 10.1016/j.brainres.2011.02.037 21338583

[B64] Yang L. Sun M. Sun X.-m. Cheng G. Z. Nicosia S. V. Cheng J. Q. (2016). Akt Attenuation of the Serine Protease Activity of HtrA2/Omi through Phosphorylation of Serine 212. J. Biol. Chem. 291 (43), 22843. 10.1074/jbc.A116.700445 27825081 PMC5077218

[B65] Yang Q.-H. Church-Hajduk R. Ren J. Newton M. L. Du C. (2003). Omi/HtrA2 Catalytic Cleavage of Inhibitor of Apoptosis (IAP) Irreversibly Inactivates IAPs and Facilitates Caspase Activity in Apoptosis. Genes Dev. 17 (12), 1487–1496. 10.1101/gad.1097903 12815069 PMC196079

